# Account of the Successful Treatment of a Large Swelling of the Lower Jaw, with an Abscess in the Neck, Occasioned by Supernumerary Teeth

**Published:** 1800

**Authors:** Thomas Whately

**Affiliations:** Member of the Corporation of Surgeons of London.


XVII. *Account of the fuccefsful Treatment of a large Swelling of the lower Jaw, with an Abfcefs in the Neck, occafioned by fupernumerary Teeth. By the fame.*

JOHN ROSE, a mulatto boy, five years and a half old, at No. 8, Red Lion Paffage, Red Lion Square, applied to me in July, 1799, for the cure of a complaint in the left fide of the lower jaw. A fmall, hard, and indolent fwelling, for which no caufe could be affigned, had appeared, about twelve months prior to this application, on the angle of that jaw, and on the lower part of the cheek. It increafed flowly in fize, but was neither much inflamed, nor very painful. But in about three months from its firft appearance, the fwelling became very large and hard, and affected nearly the whole whole cheek on that ſide; ſtill, however, without any external inflammation.

It continued nearly in this ſtate for nine months; at the expiration of which time, ſome fetid matter iſſued daily into the patient's mouth. From this period, till I ſaw him, the diſeaſe continued without any variation. Fomentations and poultices, with a variety of internal medicines, had been reſorted to during the laſt three months, but without benefit. Perceiving the matter to have that peculiar foetor which proceeds from a carious bone, I examined every part of the affected ſide of the mouth with great care, in order to find the orifice from whence it flowed; but for a long time I ſearched in vain. Of the temporary molares, the firſt only had appeared through the gum on that ſide of the jaw. The gum beyond this tooth was entire, and perfectly ſound; for the ſwelling of the cheek and jaw did not appear to have extended to the gum. The matter flowed into the mouth, in a ſmall quantity, upon every motion of the jaw; but ſuch was the difficulty of finding the orifice, (from its concealed ſituation, and the unſteadineſs of the child,) that I was, at leaſt, half an hour in afcertaining, whether it proceeded from between the jaw and the cheek, or from the infide of the jaw next the tongue, although I repeatedly wiped the mouth as dry as I could, and took every method I could think of for this purpofe. At length I was certain that the matter flowed from an orifice at the further end of the mouth, and in the moft depending part of the infide of the lower jaw, below the root of the coronoid procefs. But even after this difcovery, the fretful difpofition of my little patient, who would not fuffer me to prefs his tongue to one fide for a moment, prevented my feeing the orifice. I then put the fore-finger of my left hand upon the fpot from which I fuppofed the matter to iffue, and thought that I felt a little opening, but nothing like a carious bone or a tooth ; indeed the orifice was fo fmall, and the membrane of the mouth fo fmooth in that part, that it was with the utmoft difficulty I could afcertain any difference to the touch, between this fpot and the adjoining parts. Guided by the finger of my left hand, held over this opening, I introduced a probe, and immediately perceived the end of it to touch fome bare bone. I had no doubt, from the length of time the difeafe ease had continued, but that this bone must be exfoliated; by means of a pair of fine pointed forceps, I extracted what I took for an exfoliated bone, but it proved to be one of the molares. Through the same opening another of these teeth came out, spontaneously, immediately afterwards. I then searched with the probe for more teeth or bone, but I could feel nothing more of the kind.

Thinking that the swelling of the jaw would now subside, and that a complete cure would soon follow, I left my patient to the operations of nature; but at the end of a month, finding that the external swelling was far from being so much reduced as I expected, and that matter still issued from the orifice, I again introduced the forceps and extracted another of the molares. The swelling of the jaw continued still, though I could not find any thing more within the ulcer, and the gum and alveolar processes appeared to be perfectly sound. Shortly after this, an external inflammation, with a painful swelling, appeared between the angle of the lower jaw and the neck, a little below the ear. In defiance of every method to which I had recourse, this inflammation terminated at the expiration of a month, in in a large ſuppuration ; but I was diſappointed in the hope which I had entertained, that this abſceſs would finally remove the complaint. The ſwelling and inflammation around the abſceſs, in ſome degree, ſubſided ; but the induration remained, and at the end of two months from the burſting of the abſceſs, the ſore continued open, and had a ſcrophulous appearance. The orifice in the mouth remained alſo open, and a ſmall quantity of matter ſtill iſſued from it ; but I could not find either tooth or exfoliated bone within the cavity of the ulcer. At another trial, however, which I made about a fortnight afterwards, I was more ſucceſsful, and extracted from the ulcer within the mouth, (which had now enlarged itſelf a little,) another of the molares, and in the courſe of three or four days afterwards, about a dozen pieces of the alveolar proceſſes, ſeveral of which were of the ſize of a ſmall horſe bean ; and with theſe a thin ſhell, or the baſis of a tooth, which had began to oſſify upon its pulp.

In about a month after the extraction of the laſt tooth, and the exfoliated bones, the ſwelling of the jaw had very much ſubſided, and the abſceſs in the neck was in a fair way of being healed; ſince which it has cicatrized, and the boy is now perfectly well.

From an attentive examination of the teeth in the other parts of the jaws of this boy, and of the periods at which they appeared, ſome light may be obtained with reſpect to the cauſe of the preſent diſeaſe. It will be evident, that there has been conſiderable irregularity in the time of the appearance of the teeth, that ſome of the temporary ones are wanting, and that others are ſtanding very irregularly in the jaws. Theſe circumſtances will lead us to conclude that ſome deviation from the regular courſe of nature, has occurred in the diſeaſed jaw. Of this kind is the production of ſupernumerary teeth: which I conceive has been the principal if not the only ſource of the diſeaſe in queſtion.

The order in which the teeth appeared was exceedingly irregular. The two firſt inciſors of the upper jaw came when the boy was five months old; from that time no other tooth of any kind appeared through the gums till he arrived at the age of three years: he then cut the the two firſt inciſors of the under jaw. In about three or four months from this time, the two ſecond inciſors of the under jaw; and ſhortly afterwards, the two ſecond inciſors of the upper jaw.—One of the ſecond inciſors of the under jaw, and the correſponding tooth in the upper jaw, came with their fronts ſideways. At about four years old appeared the two temporary molares on the right ſide of the under jaw, but the temporary cuſpidatus of the ſame ſide in this jaw is ſtill wanting. About the ſame time came the cuſpidatus of the right ſide of the upper jaw, but though now nearly ſix years of age, he has not yet cut the two temporary molares on that ſide of the ſame jaw, from which we may fairly conclude that they are wanting. About this time too, appeared the ſecond of the temporary molares on the left ſide of the upper jaw; but this tooth lacerating the cheek by its irregular growth, was on that account extracted. The cuſpidatus, and the firſt of the molares, of the ſame ſide, are as yet wanting. On the left ſide of the lower jaw, which the reader will recollect is the diſeaſed part, there is the firſt temporary grinder only; the cuſpidatus, and the ſecond of the molares being deficient.

According to Mr. Hunter, in his excellent Hiftory of the Human Teeth, the body of the firft adult grinder, or that which occupies the place in the jaw next beyond the fecond of the temporary molares, is commonly formed in its alveolar focket, at the age of five or fix years; though it is ufually three or four years afterwards, before it cuts the gum. This tooth is one of thofe extracted by the forceps, as above related. It may be clearly diftinguifhed from the others,—by its being fomewhat larger,—by having no appearance of fangs, but the open cavities from which the two fangs arife, and—by the enamel not being fo completely formed as in an older tooth. (Fig. 1. Plate II.)

Another of the teeth, extracted from the ulcer, was the fecond of the temporary molares, or that which had not been cut through the gum of the difeafed jaw. This likewife may be eafily diftinguifhed from the reft,—by its being nearly as large in the body, as the laft defcribed tooth,—by the enamel being completely formed, and—by one of the fangs having nearly completed its growth:* the

* One of the fangs of the correfponding tooth extracted from the upper jaw, had completed its growth.

growth of the other fang being either impeded, (Fig. 2.) or reabſorbed from coming in contact with one of the ſupernumerary teeth, to be preſently deſcribed.

The ſhell of a tooth extracted from the ulcer, bears evident marks of being the beginning of one of the larger molares.* (Fig 5.) And it is highly probable that it is the ſecond of the adult molares, for that tooth, as Mr. Hunter obſerves, begins to be formed upon its pulp about the ſixth or ſeventh year. What renders this opinion the more probable is, that ſome of the exfoliated bones among which this tooth came away, appeared to be extracted from the root of the coronoid proceſs of that jaw, in which this tooth is ſituated at its early formation.

The two remaining teeth were evidently ſupernumerary. About the age of my patient, the firſt bicuſpis begins to be formed in its alveolar proceſs, under the root of the firſt of the temporary molares, though it does not cut the gum and take its place in the jaw, in the room of that tooth, until about the age of

* The ſhell, being very thin, was accidentally broken.

twelve years. It is impoffible, therefore, that either of thofe fhould be this young bicufpis; and as the firft of the temporary molares is ftill ftanding in the jaw, and is not undermined, this opinion is further ftrengthened. Nor is it poffible, that either of them fhould be the newly formed fecond bicufpis, which fucceeds to the fecond of the temporary molares, inafmuch as this tooth does not begin to be formed at fo early an age as that of my patient; befides, thefe teeth bear no refemblance in fhape to either of the bicufpides. They are evidently the complete bodies of molares, though fmaller than the natural fize of thofe teeth; and what is very remarkable, it is evident, that there would have been three fangs formed to each of them, had they remained in the jaw to have been completed; the bony arch being formed acrofs the bodies of thefe teeth, in which there are three diftinct orifices, evidently intended for the cavities of three fangs. (Fig. 3 and 4.) This circumftance is characteriftic of the molares of the upper jaw; and it feems probable, that, as both the temporary molares on the right fide of that jaw, and one of them on its left fide are wanting, as has before been remarked, thefe fupernumerary molares were the the effect of an irregular effort of nature to fupply thefe defects.

As the patient never received an external injury upon the part, and no other caufe can be affigned for this extraordinary difeafe, it can, I think, be beft explained, by fuppofing, that when the bodies of the fupernumerary teeth, and of the fecond temporary molares, came to their full growth, and were advancing in the growth of their fangs, and in the procefs of rifing upwards to make their way through the gum, they loft their periofteal connexion with the alveolar proceffes from inflammation occafioned by their crowded fituation, and thus became dead and extraneous bodies. The alveolar proceffes might likewife afterwards die, and exfoliate from the jaw, inftead of being abforbed, as is ufual when any of the teeth are removed from their connexion with them, by extraction or difeafe.

Or inflammation thus excited, might firft attack the alveolar proceffes, and deftroying their life, and attachment to the jaw bone, caufe them to exfoliate from it: in which cafe, all the teeth within this enclofure muft die alfo, and the whole together would become extraneous bodies, furrounded by living foft parts. On either of thefe fuppofitions we may fee why the procefs of the rifing of the teeth, and the fubfequent cutting of the gum, could not take place. As the whole of the gum appeared very thick and firm, we may thence conclude, that the matter arifing from the irritation of thefe extraneous bodies, found a readier outlet at the fide of the coronoid procefs of the jaw, than through the gum.

I have only further to remark, that as each tooth, whether it be a temporary, or an adult one, is known to have a diftinct alveolar procefs, and as there muft have been four or five feparate enclofures in this cafe, we can account for there being ten or a dozen different pieces exfoliated. For though thefe were all united together in the healthy ftate of the body, yet in the procefs of exfoliation, a difunion might take place where the attachments were weakeft. If on the other hand, we fuppofe them to have been all in one piece at the time of their exfoliation from the jaw bone, we can readily believe that they might afterwards be feparated, at the places of their weakeft attachment, by the mere length of time they were foaked in warm matter; for they they were more ſoft and ſpongy than in their natural ſtate, and when firſt extracted, were very putrid and offenſive.

